# Proteomic Studies on the Management of High-Grade Serous Ovarian Cancer Patients: A Mini-Review

**DOI:** 10.3390/cancers13092067

**Published:** 2021-04-25

**Authors:** Melissa Bradbury, Eva Borràs, Assumpció Pérez-Benavente, Antonio Gil-Moreno, Anna Santamaria, Eduard Sabidó

**Affiliations:** 1 Centre de Regulació Genòmica, Barcelona Institute of Science and Technology (BIST), Dr Aiguader 88, 08003 Barcelona, Spain; mbradbury@vhebron.net (M.B.); eva.borras@upf.edu (E.B.); 2 Department of Experimental and Health Sciences, Universitat Pompeu Fabra, Dr Aiguader 88, 08003 Barcelona, Spain; 3 Biomedical Research Group in Gynecology, Vall d’Hebron Institut de Recerca, Vall d’Hebron Barcelona Hospital Campus, Universitat Autònoma de Barcelona, Passeig Vall d’Hebron 119-129, 08035 Barcelona, Spain; asperez@vhebron.net (A.P.-B.); agil@vhebron.net (A.G.-M.); 4 Gynecologic Oncology Unit, Department of Gynecology, Hospital Universitari Vall d’Hebron, Vall d’Hebron Barcelona Hospital Campus, Passeig Vall d’Hebron 119-129, 08035 Barcelona, Spain; 5 Centro de Investigación Biomédica en Red (CIBERONC), Instituto de Salud Carlos III, Avenida de Monforte de Lemos 3-5, 28029 Madrid, Spain; 6 Cell Cycle and Cancer Laboratory, Biomedical Research Group in Urology, Vall Hebron Institut de Recerca, Vall d’Hebron Barcelona Hospital Campus, Universitat Autònoma de Barcelona, Passeig Vall d’Hebron 119-129, 08035 Barcelona, Spain

**Keywords:** ovarian cancer, mass spectrometry, proteomics, genomics, cancer tissue, biomarker

## Abstract

**Simple Summary:**

In this manuscript, we review the management strategies available for high-grade serous ovarian cancer patients and the molecular advances that are helping improve our understanding of the tumor and its response to treatment. We emphasize the role that proteomics is now playing in the study of ovarian cancer tumors and how its integration with genomics can guide the development of new biomarkers and therapeutic targets.

**Abstract:**

High-grade serous ovarian cancer (HGSC) remains the most common and deadly subtype of ovarian cancer. It is characterized by its late diagnosis and frequent relapse despite standardized treatment with cytoreductive surgery and platinum-based chemotherapy. The past decade has seen significant advances in the clinical management and molecular understanding of HGSC following the publication of the Cancer Genome Atlas (TCGA) researchers and the introduction of targeted therapies with anti-angiogenic drugs and poly(ADP-ribose) polymerase inhibitors in specific subgroups of patients. We provide a comprehensive review of HGSC, focusing on the most important molecular advances aimed at providing a better understanding of the disease and its response to treatment. We emphasize the role that proteomic technologies are now playing in these two aspects of the disease, through the identification of proteins and their post-translational modifications in ovarian cancer tumors. Finally, we highlight how the integration of proteomics with genomics, exemplified by the work performed by the Clinical Proteomic Tumor Analysis Consortium (CPTAC), can guide the development of new biomarkers and therapeutic targets.

## 1. Introduction

High-grade serous ovarian cancer (HGSC) is the most common and deadly subtype of ovarian cancer [1]. It is characterized by its late diagnosis and frequent relapse despite standardized treatments with cytoreductive surgery and platinum-based chemotherapy, and more recently the introduction of targeted therapies with anti-angiogenic drugs and poly(ADP-ribose) polymerase (PARP) inhibitors in specific subgroups of patients [2]. The past decade has seen great advances in the molecular understanding of HGSC following the publication by the Cancer Genome Atlas (TCGA) researchers. These genomic and transcriptomic findings highlighted the complexity and heterogeneity of these tumors but have not proven to be sufficient to understand tumor behavior and translate into improved clinical outcomes. For this reason, the study of proteins and their post-translational modifications are now being assessed to provide novel insights into the tumor biology and phenotypes of HGSC. In this paper, we review the proteomics studies available on patient tissue samples that have the potential to improve the management of HGSC patients through a better understanding of the tumor mechanisms, and the identification of therapy response and prognostic biomarkers in patient tissues samples. We also provide an overview of the current clinical management strategies available for HGSC patients and the most relevant advances made in the field of genomics that has preceded the advent of proteomics in ovarian cancer research.

## 2. Current Therapeutic Management of High-Grade Serous Ovarian Cancer Patients

**Figure 1:**
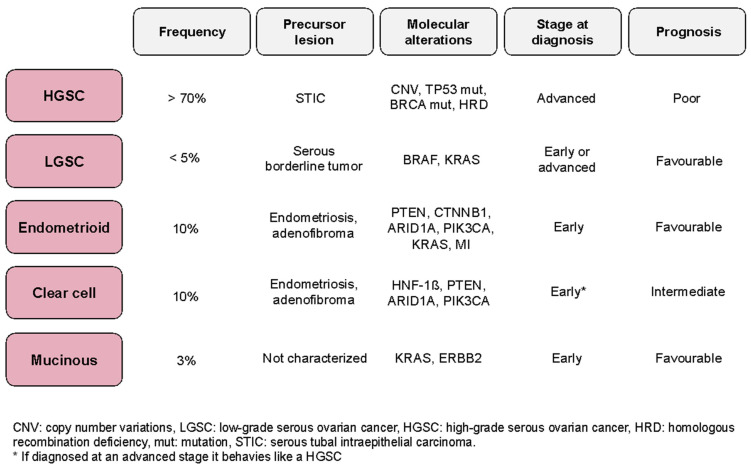
Main characteristics of the different epithelial ovarian carcinoma histological subtypes [3]. Data reflects the most common molecular alterations, stage at diagnosis, and prognosis.

Epithelial ovarian cancer (EOC) is the 8th most common cancer in women and the 7th leading cause of cancer mortality in women worldwide [1]. Although ovarian cancer is rare, it has the highest death-to-incidence ratio among all gynecological malignancies [2]. The overall 5-year survival rate is approximately 46% but varies greatly based on the histological type and stage at initial diagnosis. EOC is divided into different tumor subtypes, which differ in their etiology, histopathology, clinical presentation, molecular biology, and response to treatment (Figure 1). The five main subtypes of EOC are high-grade serous carcinomas (>70%), endometroid carcinomas (10%), clear cell carcinomas (10%), mucinous carcinomas (3%), and low-grade serous carcinomas (<5%) [3]. Of these, high-grade serous carcinoma (HGSC) is the most common and deadly subtype. While the overall survival of patients with the early-stage disease is 92%, this is reduced to 25% in patients diagnosed with advanced stages, when the disease has spread into the peritoneal cavity or to distant organs [4]. Diagnosis at advanced stages occurs in over 70% of patients due to the lack of effective screening strategies and the non-specificity of presenting symptoms which might initially be missed or attributed to other disease processes [5]. Serum CA125, HE4, and the Risk of Ovarian Malignancy Algorithm (ROMA) score are the most widely used serum markers in the diagnostic workup of patients with suspected malignant pelvic mass [6]. However, the lack of highly sensitive and specific biomarkers for the early diagnosis of pelvic mass malignancy remains an unmet clinical need. Efforts are being made to identify diagnostic biomarkers of clinical utility in biofluid specimens using proteomics approaches [7,8]. Despite its interest, this research topic is out of the scope of this review.

HGSC is thought to originate from precursor epithelial lesions in the fimbriated end of the fallopian tube although a proportion of HGSC presents without fallopian tubal involvement. It seems TP53 mutations may be an early event in the genesis of a proportion of HGSC occurring in p53 signature foci in the distal fallopian tube and the development of serious tubal intraepithelial lesions (STIC) [9]. HGSC is molecularly characterized by presenting high chromosomal instability, copy number variations, TP53 mutations. In addition, almost half have defects in the HR pathway, mainly associated with mutations in the BRCA1 and BRCA2 genes [10].

**Figure 2:**
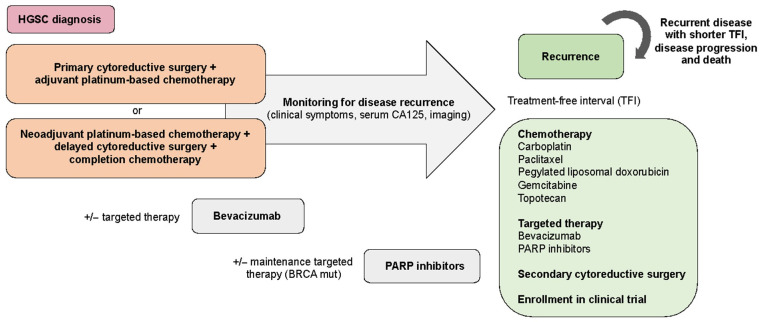
Clinical management of high-grade serous ovarian cancer at diagnosis and at the time of disease recurrence.

Tumor staging is based on the surgical assessment and pathological evaluation of cancer at the time of surgery following standardized criteria by the International Federation of Gynecology and Obstetrics (FIGO) [11]. The combination of cytoreductive surgery and platinum-based chemotherapy remains the standard of care for patients with newly diagnosed advanced-stage HGSC (Figure 2). The primary goal of surgery is to achieve complete macroscopic resection of the disease, as this has been proven to be the most important independent prognostic factor [12,13]. Cytoreductive surgery often involves extensive and complex abdominal and upper-abdominal procedures reason why careful evaluation of patients before surgery is essential for defining the best management approach [14,15]. If resection of all macroscopic diseases can be obtained based on preoperative assessment with acceptable operative morbidity, upfront cytoreductive surgery followed by platinum-based chemotherapy is the standard of care [16]. Several first-line adjuvant systemic chemotherapy strategies have led to an improvement in overall survival for patients with newly diagnosed advanced-stage HGSC. These include the addition of paclitaxel to platinum-based agents, the use of intraperitoneal chemotherapy following complete tumor resection, and the incorporation of dose-dense weekly paclitaxel treatment [17,18,19]. At present, the most widely used chemotherapy regimen is carboplatin and paclitaxel given every three weeks for six cycles. Neoadjuvant chemotherapy is an alternative treatment when the cancer burden is too extensive to allow macroscopic complete resection or for patients who are too ill for primary surgery [15]. Two trials have demonstrated comparable outcomes for first-line surgery with adjuvant chemotherapy compared with neoadjuvant chemotherapy followed by delayed surgery and completion chemotherapy [20,21].

Despite effective treatment, ~75% of women with HGSC will develop recurrence and ultimately succumb to their disease [4]. Although most recurrent HGSCs will initially respond to second-line chemotherapy, response rates diminish substantially with subsequent lines of treatment, and eventually, the disease will become resistant. The most widely used and accepted clinical surrogate of chemotherapy response is the treatment-free interval (TFI). The TFI is also used for the selection and stratification of patients for clinical trials [22]. Tumors considered to be sensitive to chemotherapy, usually based on a TFI above 6 months, can benefit from the re-use of a platinum-based doublet regimen. Various combination therapies are being used in this setting including carboplatin with paclitaxel, pegylated liposomal doxorubicin, gemcitabine, bevacizumab, or trabectedin [23,24,25]. In addition, maintenance therapy with PARP inhibitors is also being used in BRCA mutated and homologous recombination deficient (HRD) tumors. Tumors that are resistant to first-line chemotherapy (~15%) have a poor prognosis. Due to the short expected survival, usually less than 12 months, treatment is focused on quality of life and control of symptoms. Several agents including gemcitabine, weekly paclitaxel, bevacizumab, pegylated liposomal doxorubicin, and topotecan can be used as second-line treatment in these patients with modest responses [26,27]. Secondary cytoreductive surgery can be considered for patients with long TFI with a recurrence that is limited and isolated. Results from the AGO DESKTOP III study demonstrated improved progression-free survival (PFS), OS and a longer time to first subsequent therapy in patients with first recurrence undergoing secondary cytoreductive surgery with complete tumor resection [28].

Monitoring of disease recurrence is usually performed by clinical examination, imaging techniques, and serum CA125 levels. However, the use of these examinations is widely variable amongst different gynecological oncology society guidelines. Increasing levels of CA125 are generally not sufficient to initiate treatment unless a patient is symptomatic or is enrolling in a clinical trial. In addition, some patients with recurrence may not present with increased levels of CA125. Alternative markers such as HE4 are also being evaluated for the monitoring of disease recurrence and progression [29]. There are currently no markers approved in the clinic for the prediction of treatment response and the prognosis of HGSC (Table 1) [30]. 

In addition to the chemotherapy regimens used in the first and second-line setting, several targeted therapies are being investigated for the management of HGSC. Antiangiogenic therapy and PARP inhibitors are the only targeted therapies currently approved for the treatment of HGSC in the maintenance and recurrent setting. Inhibition of tumor angiogenesis with bevacizumab, an anti-vascular epithelial growth factor (anti-VEGF) monoclonal antibody, has proven to be effective for the treatment of advanced-stage HGSC. Several randomized-controlled trials have shown improved PFS rates when used in addition to standard chemotherapy and platinum-sensitive and platinum-resistant recurrent disease [27,31,32,33]. The use of bevacizumab as an addition to carboplatin and paclitaxel chemotherapy and maintenance therapy in patients with newly diagnosed advanced ovarian cancer was approved based on improvements in PFS in the ICON7 and GOG 218 studies. However, improvements in overall survival (OS) have been harder to demonstrate and are currently limited to a retrospective analysis of high-risk patients within the ICON7 trial [30]. Other molecular agents targeting angiogenesis are in preclinical and clinical development including cediranib and trebananib, which have also been assessed in combination with other targeted treatments [34].

PARP inhibitors are rapidly expanding and transforming the treatment of HGSC. These drugs target the PARP family of enzymes, principally PARP1, which play a major role in DNA repair. PARP inhibition leads to the accumulation of single-strand breaks (SSB) by disrupting the BER pathway and also causing PARP1 trapping by inhibiting auto-PARylation and/or PARP release from the DNA [35]. These effects result in the induction of double-strand breaks (DSB), which require a functional homologous recombination (HR) pathway for their repair. In HR deficient tumors (e.g., BRCA mutated tumors), the DNA lesion cannot be repaired or it is repaired by alternative pathways that are highly error-prone leading to genomic scarring and ultimately cell death [35]. A series of randomized clinical trials have consistently demonstrated the efficacy of PARP inhibition in platinum-sensitive recurrent HGSC, with the greatest benefit seen in those with BRCA mutations, both as single-agent therapy and as maintenance in the first-line and recurrent setting [36,37,38,39,40,41,42]. The first approvals for PARP inhibitor therapy in ovarian cancer were limited to patients with pathogenic BRCA mutations. Their use was later extended to patients with non-germline BRCA mutated, HRD and patients without a demonstrable defect in HR after evidence also showed a clinical benefit [38,39].

Finally, multiple trials are now assessing the combination of PARP inhibitors with other targeted therapies such as bevacizumab [43]. In addition, new DNA damage repair targets are being evaluated to overcome platinum and PARP inhibitor resistance (i.e., ATR, CHK1, and WEE1 inhibitors) [44,45,46]. Immune checkpoint inhibition has yielded impressive clinical responses in other cancers such as melanoma and non-small cell lung cancer [47,48]. Nevertheless, their use in recurrent ovarian cancer has shown modest results with overall response rates of 10–25% [49]. Several anti-PD-L1/PD1 agents including pembrolizumab, nivolumab, atezolizumab, and avelumab are being assessed alone and in combination with other targeted agents including anti-VEGF and PARP inhibitors. To date, only pembrolizumab has received approval for its use in gynecological malignancies, specifically for tumors with microsatellite instability-high or mismatch repair deficiency after progression with prior treatment [50].

It is therefore clear that a better understanding of HGSC tumor mechanisms is still required beyond current genomic knowledge to open new therapeutic opportunities and improve patient management. To achieve this, the incorporation of proteomics into ovarian cancer research is playing a crucial role to determine distinct HGSC phenotypes associated with patient clinical outcomes and the stratification of patients for adequate diagnosis and prediction of treatment response.

## 3. Tumor Mechanisms and Identification of Molecular Therapeutic Targets in HGSC

Large-scale sequencing aimed at elucidating the key genomic changes occurring in different types of cancer have allowed the generation of large comprehensive genomic data. The Cancer Genome Atlas (TCGA) and the International Cancer Genome Consortium (ICGC) have sequenced thousands of tumors across different cancer types allowing the identification of novel specific genomic profiles that are potentially clinically targetable [10]. TCGA analysis of 489 tumors revealed that HGSC is characterized by presenting high chromosomal instability and copy number alterations [10]. Almost all HGSC have a TP53 mutation and almost half have defects in the HR pathway [10]. These defects are associated with germline, somatic, and epigenetic BRCA mutations, as well as alterations in other DNA repair genes (i.e., RAD51C, RAD51D, BRIP1, MLH1, MSH2, and MSH6) [51,52]. The molecular characteristics of the other half of HGSCs (i.e., those that do not have apparent defects in HR) are less defined. Somatic copy number alterations, promoter methylation events, amplification of CCNE1 and defective Notch, PI3K, RAS-MEK, and FOXM1 signaling pathways have been identified in some HGSC [10]. Other less common mutated genes have also been reported including CSMD3, CDK12, FAT3, GABRA6, NF1, and RB1, affecting less than 5% of tumors [10]. Four molecular subtypes of HGSC have been identified by gene expression profiling and classified as immunoreactive, proliferative, differentiated, and mesenchymal types [51]. These molecular subtypes are associated with differential clinical outcomes and microenvironmental characteristics, such as immune and stromal cell activation. Despite the promising results derived from this molecular classification for the understanding of HGSC heterogeneity, it has not yet proven to be useful for patient management and has therefore not been incorporated into the clinical setting [53]. A gene-set assay is currently being evaluated to provide a gene-based molecular subtype classification of HGSC to improve classification for molecularly targeted trials [54].

Although genomic analyses have greatly improved our knowledge of HGSC, it is now clear that the molecular mechanisms of HGSC are not fully captured by genomic and transcriptomic analysis [55], thus requiring the acquisition of additional layers of information. The information provided by genomics and transcriptomics is limited to providing risk estimates, disease diagnosis, and guiding therapeutic management. However, they do not embrace the heterogeneity available in protein translation and protein post-translational and structural modifications which provide a more precise representation of the molecular tumor phenotype. Therefore, the investigation of proteins and their post-translational modifications (PTMs) are now emerging to provide novel insights into the tumor biology of HGSC. PTMs (i.e., phosphorylation, ubiquitination, acetylation, glycosylation, etc.) interplay in the modulation of protein functions thus providing information on kinase activity, the dynamic regulation of protein interactions, and cellular signaling networks [56]. Their exploration in HGSC tissues is already proving essential to advance towards a better understanding of disease mechanisms, the identification of new drug targets, and to provide new tools for the identification of predictive and prognostic markers that can help improve patient care [57,58].

Mass spectrometry (MS) is currently the technology of choice for large-scale proteomic analysis allowing proteins to be analyzed rapidly, accurately, and with high sensitivity [59]. The recent technological developments in instrumentation, sample preparation, and data analysis have resulted in the availability of high-quality, reproducible and comprehensive data from a variety of clinical samples [60,61]. The Clinical Proteomic Tumor Analysis Consortium (CPTAC) is already integrating proteomic and phosphoproteomic data with genomic data available from the TCGA for the characterization of a variety of human tumor samples, allowing for better patient stratification, and improved sensitivity to identify cancer-related pathways and potential therapeutic targets [62,63,64,65,66]. They also provide optimized workflows with higher throughput and reproducibility for global proteome and phosphoproteome analysis of patient samples [67]. They have also defined three specific tiers of targeted MS measurements for proteomics experiments according to the experimental design parameters and assay characteristics to ensure that the tests reported meeting the required levels of performance and reproducibility [68]. More importantly, the CPTAC has made significant progress in streamlining protein biomarker studies by developing accurate and reproducible quantitative proteomic workflows to verify protein biomarker candidates using targeted proteomic analyses [66,67]. These workflows have allowed us to overcome some of the handicaps of past proteomics studies and aid in the development of successful biomarkers and their translation into the clinical setting. This is essential in ovarian cancer research in which few markers have moved beyond validation and even fewer have received approval from regulatory agencies. The CPTAC has also generated well-characterized reagents for assay validation and an open-access policy for proteomic data sets. The use of proteomic approaches in the initial steps of the biomarker pipeline enables the systematic interrogation of proteomes from complex clinical samples without the need to rely on the availability of high-quality antibodies and their cross-reactivity, a limitation of commonly used immunoassays.

Several studies have analyzed protein changes in ovarian cancer cell lines for the investigation of signaling events, cellular perturbations, and response to therapies [69,70,71]. Proteomics is helping us to decipher these cellular processes to advance our understanding of HGSC disease mechanisms. The most comprehensive and integrative study aimed to molecularly characterize 26 ovarian cancer cell lines, HGSC tumors, immortalized ovarian surface epithelial cells, and fallopian tube epithelial cells [70]. Authors identified a 67 protein-based signature able to stratify cell lines and tumors into epithelial and mesenchymal clusters providing new insights into the origin of HGSC and new potential drivers of tumorigenesis [70]. Integrative proteomic analysis of formalin-fixed paraffin-embedded (FFPE) tumor samples recently identified CT45 as a novel protein phosphatase 4 regulator linked to DNA damage signaling and responsible for enhancing chemosensitivity in metastatic HGSC [71]. Comparison of 26 EOC tumors and benign samples were also evaluated for the identification of potential therapeutic targets [72]. Authors identified chloride intraepithelial channel 1 (CLIC1) and lectine galactoside-binding soluble 3 binding protein (LGALS3BP) as being overexpressed in EOC compared to normal ovarian tissues which could play a role in ovarian tumorigenesis.

Beyond proteome characterization, several studies have used proteomic approaches to specifically study protein PTM events in ovarian cancer tissues, including phosphorylation, glycosylation, and ubiquitination events, among others. The study of PTMs is essential to obtain functional information on the dynamic regulation of tumor signaling networks. Although it is now clear that HGSC is a separate subtype of EOC with unique molecular and clinical features, many studies to date have failed to make this distinction. When describing these studies we refer to EOC when, in addition to HGSC, other subtypes have been included in the analysis. Several studies have been performed by the CPTAC and other authors to provide a deeper understanding of HGSC tumors [57,58,73,74]. The largest and most comprehensive study of protein PTMs to date was performed by the CPTAC. This study integrated the genomic and transcriptomic data from the TCGA ovarian cancer cohort with the study of the proteome and phosphoproteome in 169 HGSC tumors [57]. Distinct subgroups of tumors were identified based on changes in their protein abundances, and the phosphoproteome analysis identified signaling pathways associated with differences in patient survival. With this information, the authors developed protein signatures of chromosomal instability (CIN) and HRD for patient stratification. More recently, the CPTAC has published a comprehensive proteogenomic and phosphoproteomic analysis of 83 HGSC tumors and 20 fallopian tube precursor samples [58]. The authors did not only confirm the previous TCGA findings in relation to HRD and histone acetylation in prognosis and survival but also identify mitotic and cyclin-dependent kinases as potential therapeutic targets. Tissue-derived cell lines have also been used to evaluate the proteome and phosphoproteome of EOC [75]. Authors uncovered cancer-specific kinase signatures and identified cyclin-dependent kinase 7 (CDK7) as a key regulator of EOC proliferation through the phosphorylation of polymerase II alpha (POLR2A). Proteomic quantitative analysis has also been employed to study the mitochondrial phosphoproteome in 8 EOC and 11 benign ovarian tissues, thus providing novel insights into mitochondrial protein phosphorylations and their potential roles in ovarian cancer pathogenesis [76].

Besides phosphorylation, TCGA tumor samples have also been employed to study other PTMs and their role in HGSC pathogenesis. Protein glycosylation has also been assessed in 119 samples to investigate its role in the heterogeneity of HGSC [77]. The authors used two glycoproteomic MS-based strategies for glycosylation analysis and the identification of site-specific glycans. They observed a strong relationship between glycopeptide signatures and TCGA tumor molecular subtypes associated with patient clinical outcomes. In addition, glycoproteomics has been assessed in an independent cohort of 83 HGSC tumors and 23 non-tumor tissues with the identification of three tumor clusters regulated by specific glycosylation changes and glycosylation enzymes [78].

Finally, efforts are being made to integrate the information provided by different PTMs and their complex interplay in HGSC tissues to provide an additional level of information to the tumor signaling networks. Our group has recently performed a multi-layered analysis evaluating proteome, phosphoproteome, and ubiquitinome changes in patient-derived HGSC tumors according to their BRCA1 mutation status. Our results evidence a tight coordination between ubiquitination and phosphorylation regulatory layers and their role in major cellular processes related to BRCA1-dependent HGSC pathogenesis, including DNA repair, RNA splicing, transcription regulation, and PI3K/AKT/mTOR signaling.

## 4. Therapy Response and Prognostic Molecular Biomarkers in HGSC

In addition to understanding HGSC tumor mechanisms through the study of proteins and their PTMs, the study of proteins can also help us classify patients according to a specific response to treatment or their disease prognosis. The introduction of biomarkers for cancer therapy has allowed the traditional clinical practice to move towards a more stratified approach by adding a biomarker-associated step to assign a patient to a specific management strategy or therapy. Despite the promising advantages of moving towards a stratified medicine in ovarian cancer, there are currently no approved biomarkers available for the prediction of treatment response and disease prognosis (Table 1).

Because proteins are the biological endpoints that control the majority of biological processes in the cell and are the targets of most current drugs, great efforts are being made to find protein cancer biomarkers of clinical utility. To date, the biomarker studies available from patient samples mainly come from genomic data. However, to date, these have limited clinical applicability beyond the identification of BRCA mutations and HRD status. Numerous studies have reported that mutations in the BRCA1 and BRCA2 genes are associated with increased response rates to platinum-based chemotherapy and better survival outcomes, especially for BRCA2 mutations [51,52,79]. In addition, mutations in other HR genes including ATM, BARD1, BRIP1, CHEK1, FAM175A, MRE11A, NBN, PALB2, RAD51C, or RAD51D have a similar impact on platinum response and survival [80]. Findings from the TCGA analysis reporting four molecular subtypes of HGSC (i.e., differentiated, immunoreactive, mesenchymal, and proliferative subtypes) [51] were followed by the Classification of Ovarian Cancer (CLOVAR) study aimed at developing predictors of patient outcome based on these molecular subtypes. Authors identified a CLOVAR survival signature including 100 genes able to classify patients into good, intermediate, and poor survival groups [53]. Despite the promising results, this molecular classification has not proven to be useful for patient management in the clinical setting. Another molecular study looking at a genome-wide loss of heterozygosity (LOH) and copy number changes in HGSC to predict treatment outcome, observed patients with high LOH to be more platinum-responsive, which is in line with results observed for BRCA mutations [81]. Several genetic modifications have been associated with acquired resistance to platinum-based chemotherapy such as inactivation of the tumor suppressors RB1, NF1, RAD51B, and PTEN, the amplification of CCNE1 and NACC1, reversal of germline or somatic BRCA1 and BRCA2 mutations in individual patients, and the overexpression of the drug efflux pump MDR1 [82,83]. An association between excision repair cross-complementation group (ERCC1) polymorphism and platinum sensitivity has also been reported in a few studies with conflicting results [84,85].

A variety of proteins have been identified to predict response to treatment in ovarian cancer both from in vitro and in vivo studies [69,86]. For example, p-BAD, SYK, Notch 3, DYRK2, Snail, TRIB2, MAD1, and CDK1 have been studied in vitro as potential markers of chemotherapy response [87,88,89,90,91,92,93]. However, the validity of these markers has not been further assessed in patient samples. There is increasing awareness that the pre-existing immune status can affect the response to subsequent therapy. In ovarian cancer, TLR4, MYD88, CD44, IL8, IRF1, and CD8(+) cells have been associated with differences in response and survival [94,95,96]. In addition, acetylation and phosphorylation of STAT1 can mediate platinum response through HDAC4 [97]. In peritoneal and pleural effusions, class III β-tubulin, Aurora B and Claudin 3 expression have been associated with resistance to chemotherapy and poor patient survival, although validation of these proteins in independent cohorts of patients are not available [98,99,100].

Studies evaluating predictive protein markers in ovarian cancer tissues using proteomic approaches are scarce. A recent study compared the proteomes of platinum-sensitive and platinum-resistant HGSC using FFPE tissues from omental metastasis. CT45 was found to be overexpressed in chemosensitive tumors and associated with longer survival [70]. CT45 was also shown to be linked to DNA damage signaling and the binding of HLA-I receptors for the activation of cytotoxic T cells to promote tumor killing [71]. Data from 130 tumor tissues from the CPTAC have also been employed to predict platinum drug response using supervised machine learning methods with the involvement of ATP synthesis pathways and Ran GTPase binding [101]. A recent multiomic analysis of 30 HGSC tumors aimed to identify genomic, transcriptomic, and proteomic markers associated with clinical outcomes in patients undergoing primary surgery with complete resection (R0) and patients undergoing neoadjuvant chemotherapy with either excellent or poor response to treatment [102]. Authors identified altered signaling pathways associated with phosphorylation changes in proteins including ATP2C1, STAT3, CD44, and CDK4. A recent study also evaluated proteomic changes associated with favorable and poor PFS in a total of 12 HGSC tumors. High expression of AAT, NFKB, and PMVK correlated to favorable PFS whilst high expression of VAP1, FABP4, and PF4 were associated with poor PFS [103]. These studies contribute to the great efforts that are being made by ourselves and other groups to identify protein biomarkers able to predict the response to current therapies to improve the outcomes of HGSC patients [101,104]. These efforts are added to those being made in the field of screening and early disease diagnosis [7,8].

Finally, with the introduction of targeted therapies for the treatment of ovarian cancer, efforts are also been made to identify predictors of response to these therapies, although these are proving challenging. Angiogenic markers such as CD31 expression, microvessel density, and tumor VEGF-A levels were identified in a retrospective analysis of the GOG 218 study as potential predictive and prognostic biomarkers of response to anti-angiogenic therapy [105]. A discriminatory signature comprising mesothelin, FLT4, alpha-1 acid glycoprotein (AGP), and CA125 was also reported as potentially identifying those patients with EOC more likely to benefit from bevacizumab [106]. A possible role of combined values of Ang1 and Tie2 as predictive biomarkers for improved PFS in bevacizumab-treated patients with EOC has also been suggested [107]. With regards to PARP inhibitors, no protein markers have been identified to date for the prediction of treatment response in ovarian cancer. Potential predictive genomic markers have been associated with LOH, secondary BRCA2 mutations, and CDK12 mutations [108,109,110]. However, the validation of all these findings in the clinical setting is still required.

## 5. Conclusions

Ovarian cancer represents a heterogeneous disease with unique histological subtypes that differ in their clinical presentation, genomic profiles, response to treatment, and prognosis. HGSC is the most common and deadly subtype and the focus of most comprehensive ovarian cancer studies. Recent molecular advances have provided useful insights into HGSC tumor biology. However, these findings have not been sufficient to understand its clinical behavior and translate into improved clinical outcomes.

Proteomics provides an additional functional layer of information able to enhance our knowledge of the complex physiology of HGSC tumors, firstly, through the understanding of the tumor mechanisms and the identification of actionable therapeutic targets and, secondly, through the identification of protein biomarkers able to predict treatment response and disease prognosis. Mass spectrometric (MS) approaches enable a systematic interrogation and characterization of proteomes from complex samples with high sensitivity, precision, and sample throughput. However, the implementation of meaningful results obtained by MS technologies is currently limited by their transferability to other techniques of routine use in the clinics (i.e., antibody-based assays).

MS-based proteomics technology must move beyond its current role as a powerful research tool in specialized laboratory environments to clinical settings, in the same way as MS has been adopted in the fields of clinical microbiology and the characterization of small molecules (e.g., vitamin D, steroids). There is no doubt that proteomics will continue to provide a valuable resource to unravel the complexity of HGSC tumors and that its integration with other disciplines will be essential for improving patients’ outcomes and advance towards more personalized patient care.

## Figures and Tables

**Table 1:** List of protein biomarkers approved by the U.S. Food and Drug Administration (FDA) in ovarian cancer [30].

Biomarker	Type	Specimen	Method	Clinical Use	Year
CA125	Protein	Serum, plasma	Immunoassay	Monitoring treatment response	1997
HE4	Protein	Serum	Immunoassay	Monitoring disease recurrence or progression	2008
ROMA (HE4 + CA125)	Protein	Serum	Immunoassay	Prediction of pelvic mass malignancy	2011
OVA1 Next Generation	Protein	Serum	Immunoassay	Prediction of pelvic mass malignancy	2009

CA125: cancer antigen 125; HE4: human epididymis protein 4; ROMA: Risk of Ovarian Malignancy Algorithm; PCR: polymerase chain reaction.
